# Metabolic Interventions to Prevent Hypertrophy-Induced Alterations in Contractile Properties In Vitro

**DOI:** 10.3390/ijms22073620

**Published:** 2021-03-31

**Authors:** Ilvy M. E. Geraets, Will A. Coumans, Agnieszka Strzelecka, Patrick Schönleitner, Gudrun Antoons, Francesco Schianchi, Myrthe M. A. Willemars, Dimitrios Kapsokalyvas, Jan F. C. Glatz, Joost J. F. P. Luiken, Miranda Nabben

**Affiliations:** 1 Department of Genetics & Cell Biology, Faculty of Health, Medicine and Life Sciences, Maastricht University, 6200-MD Maastricht, The Netherlands; ilvygeraets@hotmail.com (I.M.E.G.); W.Coumans@maastrichtuniversity.nl (W.A.C.); a.strzelecka@maastrichtuniversity.nl (A.S.); f.schianchi@maastrichtuniversity.nl (F.S.); m.willemars@maastrichtuniversity.nl (M.M.A.W.); d.kapsokalyvas@maastrichtuniversity.nl (D.K.); glatz@maastrichtuniversity.nl (J.F.C.G.); j.luiken@maastrichtuniversity.nl (J.J.F.P.L.); 2 Departments of Physiology, Maastricht University, 6200-MD Maastricht, The Netherlands; patrick@ionoptix.com (P.S.); Gudrun.Antoons@UGent.be (G.A.); 3 CARIM School for Cardiovascular Diseases, Maastricht University, 6200-MD Maastricht, The Netherlands; 4 Department of Clinical Genetics, Maastricht University Medical Center, 6200-MD Maastricht, The Netherlands

**Keywords:** cardiac hypertrophy, glucose uptake, phenylephrine, metabolic modulation, adult rat cardiomyocytes

## Abstract

(1) Background: The exact mechanism(s) underlying pathological changes in a heart in transition to hypertrophy and failure are not yet fully understood. However, alterations in cardiac energy metabolism seem to be an important contributor. We characterized an in vitro model of adrenergic stimulation-induced cardiac hypertrophy for studying metabolic, structural, and functional changes over time. Accordingly, we investigated whether metabolic interventions prevent cardiac structural and functional changes; (2) Methods: Primary rat cardiomyocytes were treated with phenylephrine (PE) for 16 h, 24 h, or 48 h, whereafter hypertrophic marker expression, protein synthesis rate, glucose uptake, and contractile function were assessed; (3) Results: 24 h PE treatment increased expression of hypertrophic markers, phosphorylation of hypertrophy-related signaling kinases, protein synthesis, and glucose uptake. Importantly, the increased glucose uptake preceded structural and functional changes, suggesting a causal role for metabolism in the onset of PE-induced hypertrophy. Indeed, PE treatment in the presence of a PAN-Akt inhibitor or of a GLUT4 inhibitor dipyridamole prevented PE-induced increases in cellular glucose uptake and ameliorated PE-induced contractile alterations; (4) Conclusions: Pharmacological interventions, forcing substrate metabolism away from glucose utilization, improved contractile properties in PE-treated cardiomyocytes, suggesting that targeting glucose uptake, independent from protein synthesis, forms a promising strategy to prevent hypertrophy and hypertrophy-induced cardiac dysfunction.

## 1. Introduction

Heart failure is the leading cause of morbidity and mortality worldwide [1]. Cardiac hypertrophy accompanies many forms of heart disease and is associated with significantly increased risk of progression into failure [2,3]. Cardiac hypertrophy is characterized by an increased cardiac muscle mass due to enhanced protein synthesis [4]. The structural and functional abnormalities in the hypertrophying heart are accompanied by a metabolic switch from mitochondrial oxidative metabolism to an increase in glucose uptake and glycolysis [5,6]. It has been proposed that these derangements in cardiac energy substrate metabolism play a key role in the pathogenesis of cardiac hypertrophy and eventually heart failure [7]. However, the exact mechanism and time course of how these metabolic abnormalities relate to changes in protein synthesis and how they contribute to the development of contractile dysfunction remain unknown. 

Chronic adrenergic stimulation plays a central role in the pathobiology of cardiac hypertrophy and failure. The α1-adrenoceptor agonist phenylephrine (PE) has previously been shown to elicit a marked hypertrophic response in vitro in neonatal cardiomyocytes, as evidenced by a significant increase in cell surface area, enhanced protein synthesis, and an increase in expression of hypertrophic markers such as atrial natriuretic factor (ANF) and brain natriuretic peptide (BNP) [8,9,10,11]. The increase in protein synthesis is most likely due to activation of mammalian target of rapamycin complex-1 (mTORC1), which is a central regulator of protein synthesis and therefore an important contributor to the development of cardiac hypertrophy [12,13]. The increased expression of maladaptive hypertrophic markers is regulated by another pathway, and starts with the well-known activation of members of the protein kinase-C (PKC) family [14], resulting in activation of protein kinase-D1 (PKD1). Subsequently, PKD1 phosphorylates histone deacetylase 5 (HDAC5). In its non-phosphorylated state, HDAC5 binds to and inactivates the hypertrophic transcription factor MEF2. Upon HDAC5 phosphorylation, MEF2 is released, enabling this transcription factor to migrate to the nucleus to start the hypertrophic gene program [15]. 

The PE-mediated hypertrophic response in vitro is similar to the in vivo changes seen in the hypertrophic heart. However, the effect of PE stimulation on glucose uptake [11,16,17,18,19,20,21,22] and on contractile function [23] has not been investigated in an integrative manner. Moreover, neonatal cardiac myocytes may not be the preferable cell model to investigate the mechanisms underlying the development of hypertrophy in the adult heart. Yet, in contrast to neonatal cardiomyocytes, PE stimulation of adult cardiomyocytes has been rarely applied for studies of cardiac hypertrophy and failure. Therefore, the first aim of this study was to investigate whether PE-treatment in adult rat cardiomyocytes depicts the well-characterized metabolic (increased glucose uptake), structural (cardiac hypertrophy), and functional (contractile alterations) abnormalities of the hypertrophic heart. Secondly, the development of structural, metabolic, and functional changes were investigated over time. We used adult rat cardiomyocytes rather than neonatal cardiomyocytes or cardiac cell lines because energy metabolism in these latter two cell types is mainly driven by glycolysis, whereas adult rat cardiomyocytes predominantly use fatty acids as the substrate for oxidative energy provision similarly to the healthy adult human heart [24].

Targeting derangements in substrate metabolism as an approach to prevent the development of cardiac hypertrophy and other types of heart failure has received considerable interest. Metabolically, the hypertrophic heart is characterized by a shift in substrate preference with an increase in glucose uptake. Consequently, fatty acid oxidation is decreased [24]. With respect to beneficial metabolic strategies, studies have shown that preservation of fatty acid oxidative metabolism during hypertrophic stimulation prevents hypertrophic growth and contractile dysfunction [22,24,26]. Moreover, the predominant glucose transporter in the adult heart, GLUT4, which facilitates glucose uptake into cardiomyocytes, has been considered a suitable target in the treatment of diabetic cardiomyopathy [27,28]. Insulin is a major stimulator of GLUT4 translocation to the sarcolemma, and insulin-stimulated GLUT4 translocation is mediated by the phosphatidylinositol-3 kinase (Pi3K)/Akt-signaling pathway, leading to phosphorylation of AS160 and de-inhibition of GLUT4-dedicated Rab proteins [27,28]. This specific pathway may therefore function as a potential target to restore the increased glucose utilization in the hypertrophic heart and prevent contractile alterations. GLUT4 translocation can also occur in an insulin-independent manner, and this requires the simultaneous activation of AMP-activated protein kinase (AMPK) and PKD1 [28]. AMPK activation serves to activate the Rab proteins via AS160 phosphorylation, similarly to insulin-stimulated GLUT4 translocation. PKD1 phosphorylates HDAC5, but this does not lead directly to GLUT4 translocation. Next to HDAC5, PKD1 also phosphorylates a lipid kinase, called phosphatidylinositol-4 kinase-iiiβ, which initiates GLUT4-vesicle budding at the endosomes [29]. Yet, Akt remains a key signaling node for regulation of GLUT4 translocation. Another downstream target of Akt is mTORC1, which regulates protein synthesis as mentioned above. Targeting this pathway could thus also prevent the hypertrophy-related structural changes in the heart. 

Next to specific inhibition of the Pi3K/Akt-pathway to target substrate uptake and protein synthesis in the hypertrophic heart, our attention was drawn by a cardiovascular drug, dipyridamole (DPY). This drug is being used in the clinic because of its vasodilating properties and its ability to inhibit platelet aggregation [30,31]. Independently of these properties, DPY directly inhibits the trans-sarcolemmal glucose transport function of GLUT4, and consequently cellular glucose uptake [32,33]. This inhibition of glucose uptake together with its Food and Drug Administration-approval makes DPY a potentially interesting compound to prevent the development of cardiac hypertrophy and hypertrophy-induced failure. Therefore, we used our in vitro model of PE-induced cardiac hypertrophy to study whether the administration of Akt-I or DPY is able to restore substrate metabolism (via inhibition of glucose uptake), thereby possibly preventing the development of structural and functional abnormalities. 

## 2. Results

### 2.1. Time-Course of Phenylephrine

The first part of this study was devoted to an in depth characterization of metabolic, structural, and functional changes in a time-dependent manner in adrenergic stimulated cardiomyocytes in transition to hypertrophy and contractile failure. 

#### 2.1.1. Effect of Phenylephrine on Hypertrophic Response

**Figure 1:**
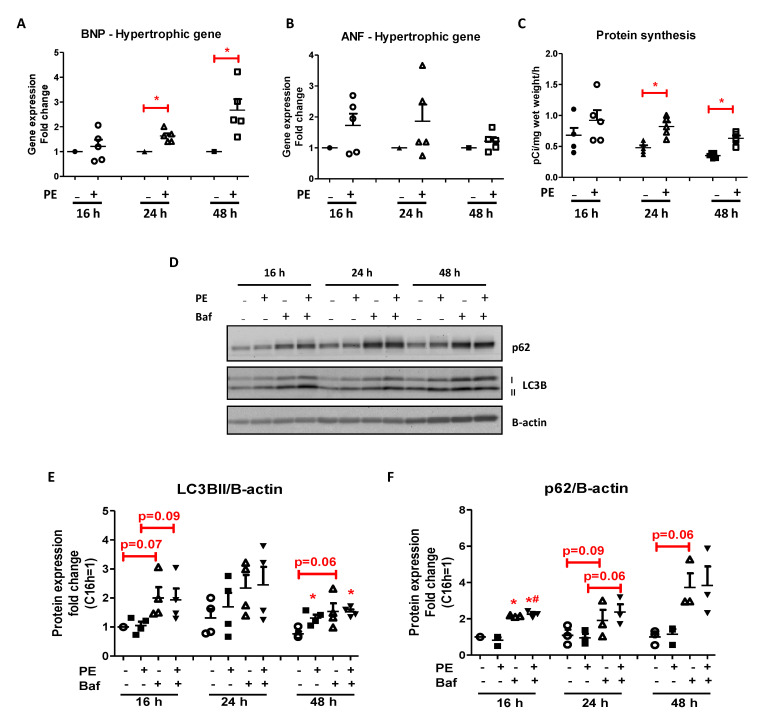
Time-dependent effects of phenylephrine on hypertrophic gene expression, protein synthesis, and autophagy. (**A**,**B**) mRNA expression levels of hypertrophic markers brain natriuretic peptide (BNP) and atrial natriuretic factor (ANF) after 16 h, 24 h, and 48 h treatment with phenylephrine (50 µM), expressed in fold increase relative to control (*n* = 5). (**C**) Protein synthesis after 16 h, 24 h, and 48 h stimulation with phenylephrine (50 µM), using [14C] phenylalanine incorporation expressed in pCi/mg wet weight/hour. Data are presented as means ± standard deviation (STDEV) (*n* = 5). (**D**) Representative Western blots of the autophagy-related proteins, LC3B and p62, after 16 h, 24 h, and 48 h phenylephrine (PE) stimulation compared to control. Co-treatment with bafilomycin was added to inhibit autophagic flux, and β-actin was used as the loading control. (**E**,**F**) Quantification of LC3BII and p62 protein levels normalized to β-actin. Values are expressed as fold increase ± STDEV relative to 16 h control incubation (*n* = 3/*n* = 4). * *p* < 0.05 compared to control, # *p* < 0.05 compared to PE were considered significantly different at the same incubation time.

Hypertrophy in adult rat cardiomyocytes was determined by measuring mRNA levels of ANF and BNP. As shown in Figure 1A, PE increased BNP expression levels after 24 h and 48 h incubation compared to that of control, but had no effect on BNP expression levels after 16 h incubation. ANF expression levels tended to be upregulated by PE after 16 and 24 h incubation and were almost back to normal after 48 h (Figure 1B). 

#### 2.1.2. Effect of Phenylephrine on Protein Turnover

To investigate the role of PE on protein turnover, we explored the effect of PE stimulation on protein synthesis and autophagy. Protein synthesis rate, as measured by amino acid incorporation, was not affected after 16 h PE stimulation, but was significantly increased after 24 h (1.7-fold, *p* < 0.05) and 48 h (1.8-fold, *p* < 0.05) compared to that of control (Figure 1C). With respect to autophagy, no difference in LC3BII and p62 protein levels were observed between control and PE cells after 16 h and 24 h, but a discrepancy between LC3BII was noted after 48 h PE stimulation (Figure 1D,E). Bafilomycin treatment tended to increase LC3BII and p62 protein levels after 16 h and 24 h incubation in both control and PE-stimulated cells to a similar extent (Figure 1D,F). This indicates that autophagy can be properly assessed in cardiomyocytes given the expected bafilomycin response, and that PE only mildly influenced autophagic flux at a late time point. The increase in protein synthesis together with an unaltered autophagic flux, which was observed after 24 h PE stimulation, was accompanied by an increased cell width/length ratio without changes in cell surface area (Supplemental Figure S1). Hence, at the morphological level, there is an alteration in cell shape without detectable change in cell size, in agreement with work by others (e.g., [34]).

**Figure 2:**
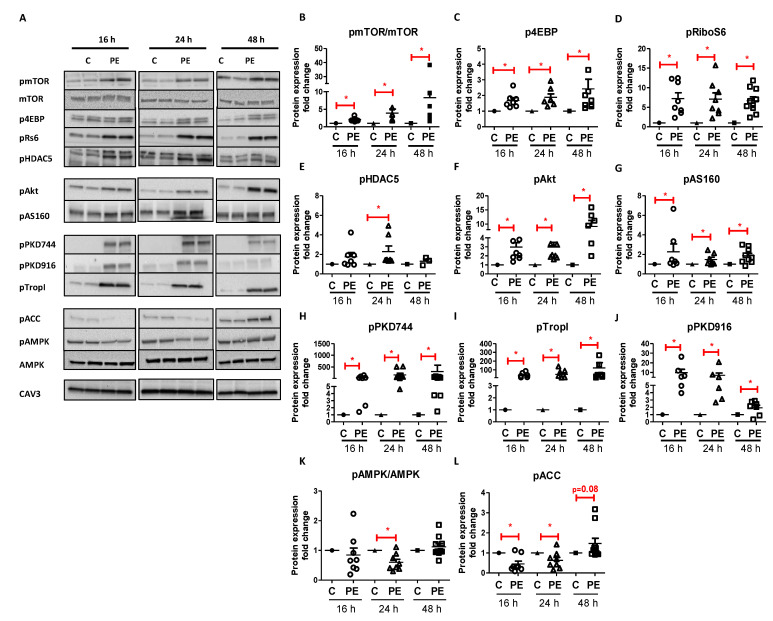
Time-dependent effect of phenylephrine on cellular signaling. (**A**) Representative Western blots showing phosphorylation levels of proteins involved in either protein synthesis (p-mTOR(ser2448), total mTOR, p-4EBP(Thr37/46), p-rS6(Ser235/236), and p-HDAC5(Ser498)) or the regulation of cellular glucose uptake (p-Akt(Ser473), p-AS160(Thr642), p-PKD1(Ser744), p-PKD1(Ser916), p-TropI(Ser23/24), p-AMPK(Thr172), total AMPK, and p-ACC(Ser79)) after 16 h, 24 h, and 48 h phenylephrine (50 µM) stimulation. (**B**–**L**) Quantification of these protein levels normalized to Caveolin 3 (CAV3). Values are expressed as fold increase ± STDEV relative to their control at the same incubation time (*n* = 7/*n* = 9). * *p* < 0.05 was considered significantly different compared to control at the same incubation time.

In order to unravel the molecular mechanism underlying the PE-induced increase in protein synthesis and ultimately cardiac hypertrophy and contractile failure, we investigated the effect of PE on the Pi3K/Akt/mTOR-pathway and on the PKD1-HDAC5 axis. Phosphorylation levels of mTOR and of its downstream targets, 4E-binding protein (4EBP) and ribosomal protein S6 (rS6), were increased after 16 h, 24 h, and 48 h stimulation with PE compared to that of control cells (Figure 2A–D). Interestingly, these signaling pathways already changed after 16 h PE stimulation and thereby preceded changes in protein synthesis and hypertrophic markers. These results indicate a possible involvement of the Pi3K/Akt/mTOR-pathway in the development of cardiac hypertrophy. Next to the increased mTORC1 activity, pHDAC5 showed an increased phosphorylation state after 24 h stimulation (not 16 h) with PE (Figure 2A,E). This suggests a possible role for the PKD1-pathway in the development of cardiac hypertrophy.

#### 2.1.3. Effect of Phenylephrine on Cellular Glucose Uptake

**Figure 3:**
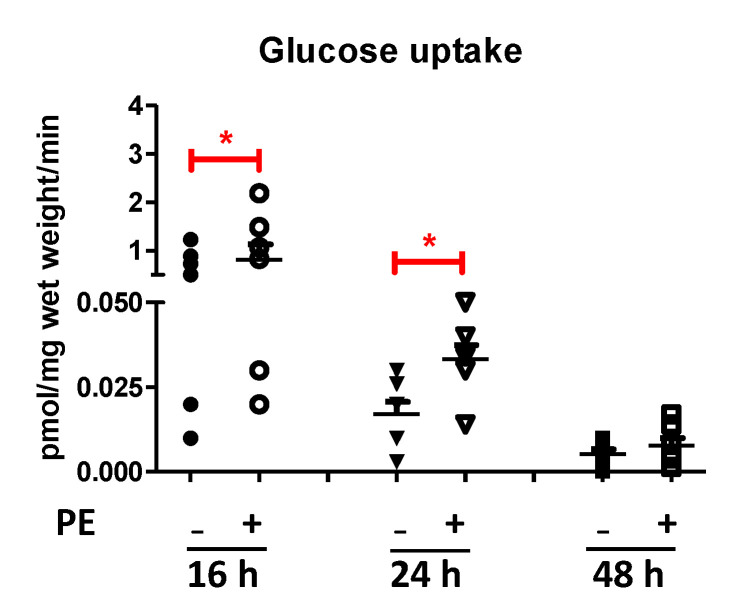
Time-dependent effect of phenylephrine on cellular substrate uptake. Glucose uptake after 16 h, 24 h, and 48 h phenylephrine (50 µM) stimulation, expressed in pmol/mg wet weight/min. Values are expressed as means ± STDEV for each incubation time (*n* = 7). * *p* < 0.05 was considered significantly different compared to control at the same incubation time.

Glucose uptake was measured in cultured cardiomyocytes via a radiolabeled assay. PE significantly increased cellular glucose uptake after 16 h (1.7-fold, *p* < 0.05) and 24 h (2.0-fold, *p* < 0.05) incubation compared to that of their controls, but no significant effect of PE was observed after 48 h (Figure 3). 

To investigate the possible mechanism leading to increased cellular glucose uptake, we evaluated the Pi3K/Akt/AS160-pathway, the PKD1-pathway, and the AMP-activated protein kinase (AMPK)-pathway in more detail, each well-known for their key regulatory roles in GLUT4 translocation [22,27]. PE stimulation resulted in an upregulation of both the Pi3K/Akt/AS160-pathway (visualized by increased degree of phosphorylation of the Akt protein and its substrate AS160) and the PKD1-pathway (visualized by increased degree of phosphorylation of PKD at ser744 and ser916, and troponin I at ser23 and ser24 (TropI)) after 16 h, 24 h, and 48 h stimulation compared to those of control (Figure 2F–J). In contrast, PE stimulation decreased AMPK phosphorylation (Figure 2A,K) after 24 h compared to that of control, without any alterations in total AMPK protein expression (Figure 2A). Approximately in line with this, acetyl-CoA carboxylase (ACC) phosphorylation was decreased after 16 h and 24 h PE stimulation compared to that of control (Figure 2A,L), suggesting a decrease in shuttling of fatty acids into the mitochondria. Taken together, these results show that stimulation of glucose uptake correlates proportionally to upregulation of the Pi3K/Akt/AS160-pathway and the PKD1-pathway.

#### 2.1.4. Effect of Phenylephrine on Contractile Function

**Figure 4:**
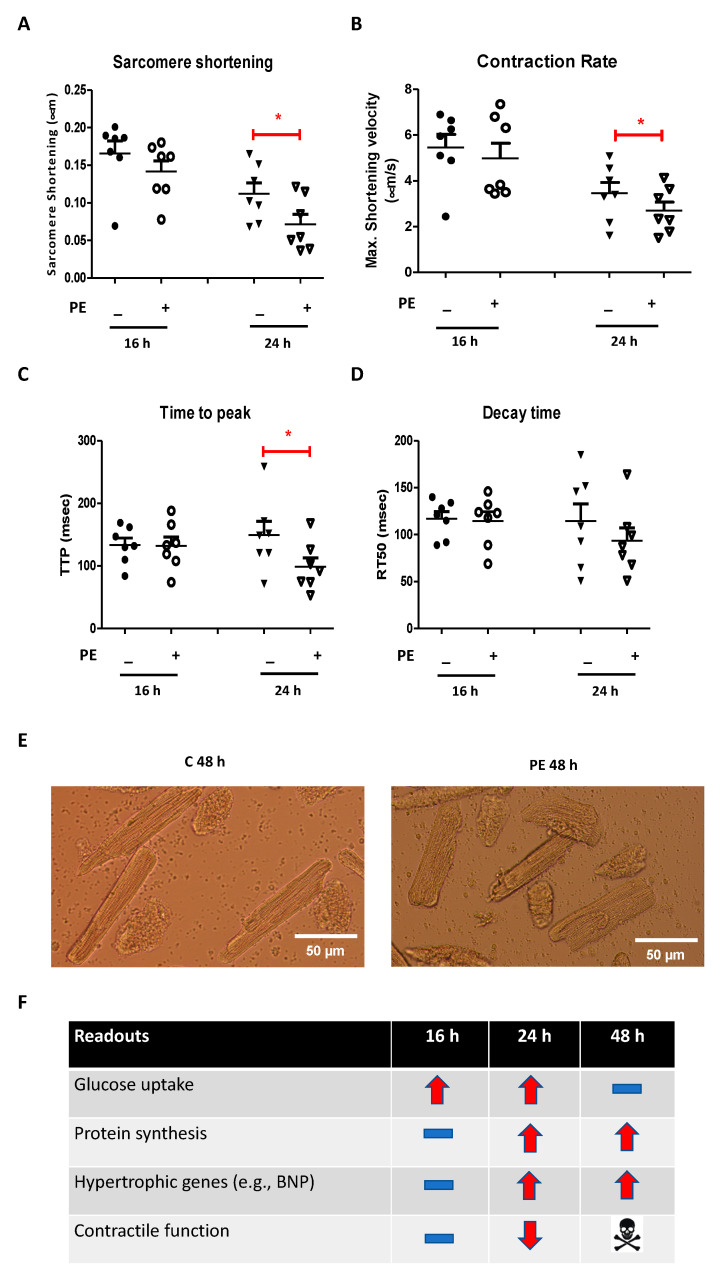
Contractile properties of isolated cardiomyocytes after 16 h, 24 h, and 48 h phenylephrine (50 µM) treatment, at 1 Hz electric-field stimulation, and a summary of the main findings with respect to the established in vitro model of cardiac hypertrophy. (**A**–**D**) Sarcomere shortening, contraction rate, time to peak, and decay time after 16 h and 24 h phenylephrine stimulation compared to that of control. (**E**) Representative pictures of control and phenylephrine-stimulated cells after 48 h culture. (**F**) Table depicting the effects of phenylephrine treatment on glucose uptake, protein synthesis, hypertrophic gene expression, and contractile function after 16 h, 24 h, and 48 h phenylephrine stimulation. The minus symbols indicate no change. The arrows up and down indicate an increase or decrease, respectively. The hazard symbol indicates that the cells died. Values are expressed as means ± STDEV for each incubation time (*n* = 7; imaging of 10 cells/condition). * *p* < 0.05 was considered significantly different compared to control at the same incubation time.

Contractile capacity of cells was determined by measuring sarcomere shortening upon 1 Hz electric field stimulation. PE stimulation for 16 h did not affect contractile function compared to that of control (Figure 4A–D), but cells stimulated with PE for 24 h showed decreased contractile function, decreased contraction rate, and reduced time to peak compared to those of control, as evident from the 35% decrease in sarcomere shortening, 22% decrease in maximal shortening velocity, and 34% decrease in time to peak (Figure 4A–C). Yet, the duration of complete relaxation remained unaffected (Figure 4D). Cells started to lose their normal shape after 48 h PE stimulation (Figure 4E), and additional exposure to an electric field caused them to die. Therefore, it was impossible to record contractile function at the 48 h time point.

### 2.2. Metabolic Interventions to Rescue Phenylephrine-Induced Contractile Alterations

Upon establishing an in vitro cell system of cardiac hypertrophy and failure, we aimed to target substrate metabolism (inhibition of glucose uptake) as an approach to prevent structural and functional abnormalities. Because 24 h was the only time point at which all tested characteristics of the hypertrophic heart (increased glucose uptake, increased protein synthesis, increased hypertrophic markers, and reduced contractile function) were apparent (Figure 4F), we focused on this specific incubation time in the second part of this study. As mentioned in the introduction, Akt-I and DPY were chosen as metabolic modulators.

#### 2.2.1. Effect of PAN-Akt Inhibitor on Phenylephrine-Induced Metabolic, Structural, and Functional Parameters

**Figure 5:**
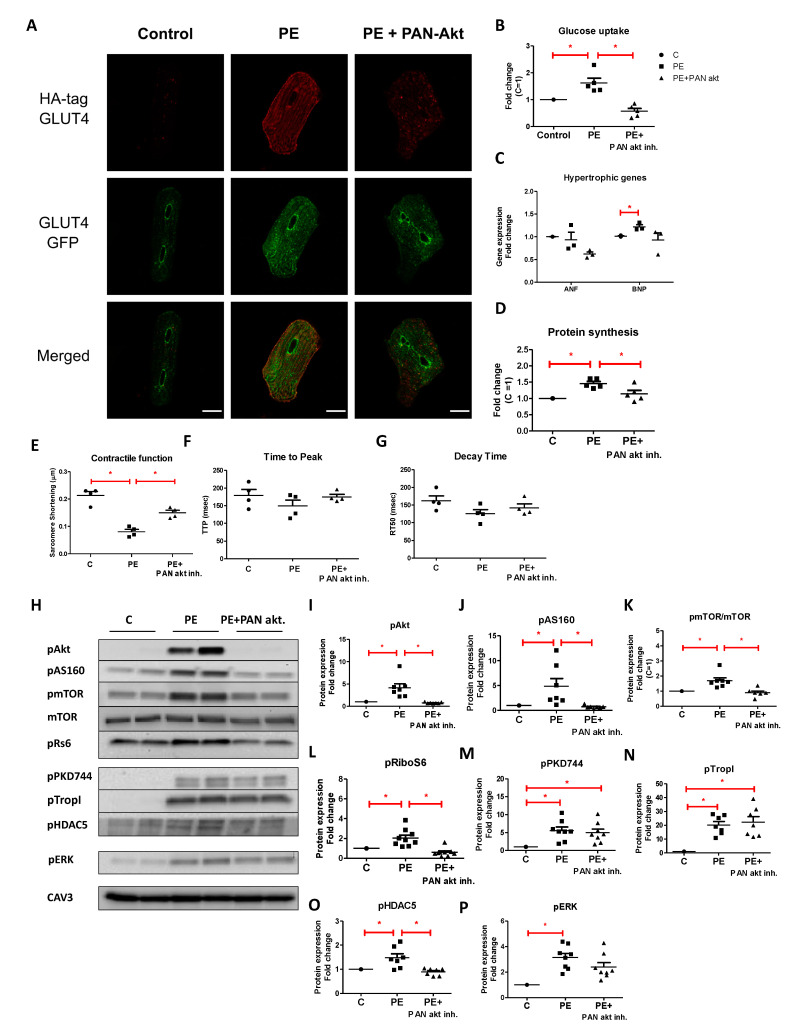
The effect of the PAN-Akt inhibitor (5 µM) on GLUT4 translocation, glucose uptake, hypertrophic gene expression, protein synthesis, contractile function, and cellular signaling after co-treatment with PE (50 µM) for 24 h. GLUT4 translocation (scale bar 20 µm) (representative confocal images of *n* = 3, see Supplemental Figure S2) (**A**), glucose uptake (*n* = 5) (**B**), hypertrophic gene expression (*n* = 3) (**C**), and protein synthesis (*n* = 5) (**D**) are displayed in fold increase relative to control. (**E**–**G**) Contractile properties upon 1 Hz electric-field stimulation: sarcomere shortening, time to peak, and decay time. Values are expressed as means ± STDEV (*n* = 4). (**H**) Representative Western blots showing phosphorylation levels of proteins involved in the Pi3K/Akt/mTOR-pathway (p-Akt(Ser473), p-AS160(Thr642), p-mTOR(ser2448), mTOR, and p-rS6(Ser235/236)) and the PKD1-pathway (p-PKD1(ser744), p-PKD1(ser916), p-TropI(Ser23/24), and p-HDAC5(Ser498)) (**H**–**O**). Finally, phosphorylation of ERK1/2 (p-ERK1/2-thr202/tyr204) is shown (**P**). For quantification, protein levels were normalized to Caveolin 3 (CAV3). Values are expressed as fold increase ± STDEV relative to control (*n* = 7/*n* = 9). * *p* < 0.05 was considered significantly different compared to control.

Twenty-four-hour treatment of PE-stimulated cells with Akt-I prevented a PE-induced increase in GLUT4 translocation from endosomes to the cell surface (Figure 5A + Supplementary Figure S2), and consequently an increase in cellular glucose uptake (Figure 5B). Akt-I also prevented increased hypertrophic gene expression (Figure 5C) and protein synthesis (Figure 5D). Moreover, Akt-I improved PE-induced contractile alterations (Figure 5E–G). 

To confirm specificity of Akt-I, the effect of Akt-I on phosphorylation levels of proteins involved in the Pi3K/Akt/mTOR-signaling cascade was explored. Indeed, Akt-I prevented a PE-induced increase in phosphorylation levels of Akt and AS160 (Figure 5H–J). Moreover Akt-I prevented phosphorylation of mTOR and rS6 (Figure 5H,K,L). These effects of Akt-I on downstream Akt signaling were to be expected. In contrast, Akt-I had no effect on the degree of phosphorylation of PKD1, TropI (Figure 5H,M,N), and ERK1/2 (Figure 5H,P), suggesting that its off-target signaling effects may be limited. Unexpectedly, however, we also observed a significant decrease in the HDAC5 phosphorylation state in cells co-treated with Akt-I when compared to that in PE-stimulated cells (Figure 5H,O), which still might be related to a downstream action of this inhibitor as explained in the Discussion.

#### 2.2.2. Effect of Dipyridamole on Phenylephrine-Induced Metabolic, Structural, and Functional Alterations

**Figure 6:**
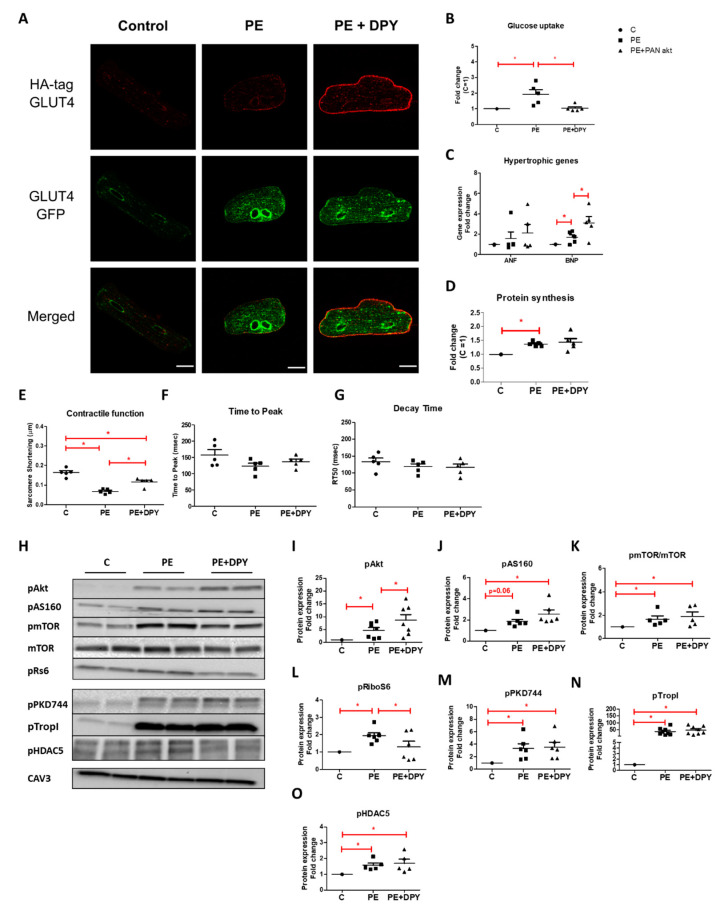
The effect of dipyridamole (DPY) (10 µM) on GLUT4 translocation, glucose uptake, hypertrophic gene expression, protein synthesis, contractile function, and cellular signaling after 24 h stimulation with phenylephrine (50 µM). GLUT4 translocation (scale bar 20 µm) (representative confocal images of *n* = 3, also see Supplemental Figure S2) (**A**), glucose uptake (*n* = 5) (**B**), hypertrophic gene expression (*n* = 5) (**C**), and protein synthesis (*n* = 5) (**D**) are displayed in fold increase relative to control. (**E**–**G**) Contractile properties upon 1 Hz electric-field stimulation: sarcomere shortening, time to peak, and decay time. Values are expressed as means ± STDEV (*n* = 5). (**H**) Representative Western blots depicting phosphorylation levels of proteins involved in the Pi3K/Akt/mTOR-pathway (p-Akt(Ser473), p-AS160(Thr642), p-mTOR(ser2448), and p-rS6(Ser235/236)) and the PKD1-pathway (p-PKD1(ser744), p-TropI(Ser23/24), and p-HDAC5(Ser498)). (**I**–**O**) For quantification, protein levels were normalized to Caveolin 3 (CAV3). Values are expressed as fold increase ± STDEV relative to control (*n* = 5/*n* = 7). * *p* < 0.05 was considered significantly different compared to control.

DPY treatment did not inhibit PE-stimulated GLUT4 translocation, but rather stimulated this process (Figure 6A, see Supplemental Figure S2). This is an unexpected finding since under control conditions (i.e., in the absence of PE), DPY did not influence GLUT4 translocation [33]. We have no explanation for this surprise finding. In contrast, DPY treatment prevented a PE-induced increase in glucose uptake (Figure 6B). Hence, the stimulatory action of DPY on GLUT4 translocation is overruled by its inhibitory action on GLUT4 transport activity. Furthermore, DPY ameliorated PE-induced contractile alterations (Figure 6E–G). Interestingly, DPY did not restore the PE-induced increase in protein synthesis and even enhanced a PE-induced increase in hypertrophic markers (Figure 6C,D).

DPY treatment led to further increased phosphorylation levels of Akt and AS160 in PE-stimulated cells (Figure 6H–J). Additionally, DPY did not prevent the PE-induced increase of mTOR activity (Figure 6H,K), but prevented the PE-induced increase in rS6 phosphorylation (Figure 6H,L). With respect to the PKD1-pathway, DPY did not influence the PE-induced increase in the phosphorylation state of PKD744, TropI, nor HDAC5 (Figure 6H,M–O). These results indicate that DPY does not elicit its inhibitory effects on glucose uptake through these specific pathways.

## 3. Discussion

### 3.1. In Vitro Characterization of Metabolic, Structural, and Functional Changes during the Development of Hypertrophy and Failure

The present study showed that 16 h adrenergic stimulation of adult rat cardiomyocytes by using PE was associated with an increase in glucose uptake only, while 24 h PE treatment led to both increased glucose uptake, enhanced protein synthesis, increased expression of hypertrophic markers, and reduced contractile function. Although 48 h PE treatment showed similar effects on metabolic and hypertrophic parameters as those at the 24 h time point, cells were too severely affected to undergo controlled contraction upon electric field stimulation. These data indicate that 24 h PE stimulation functions as a representative model to mimic cardiac hypertrophy in vitro, whereas 48 h is more representative of heart failure. 

A PE-induced hypertrophic response in isolated cardiomyocytes has been reported before. Several studies have shown that PE increases cell size, expression of hypertrophic markers, and protein synthesis rate, which form the main readout parameters of cardiac hypertrophy [4], however, most of these studies were performed in neonatal cardiomyocytes and did not include the time-course of changes. Our present study supports these findings for adult rat cardiomyocytes. Where protein synthesis was affected after 24 h PE treatment, no effect on autophagy was observed at this particular time point. This suggests that the 24 h time point reflects an early-stage of heart failure, where protein turnover is shifted towards net protein synthesis, contributing to the development of cardiac hypertrophy. 

Our observations with Akt-I suggest that the Pi3K/Akt/mTOR-pathway, via activation of the mTORC1 complex, plays an important role in the development of PE-induced cardiac protein synthesis and thus hypertrophy. Additionally, inhibition of Akt with Akt-I resulted in a decreased phosphorylation of HDAC5, which is rather surprising since Akt and HDAC5 have not yet been connected with hypertrophic signaling in the heart. Yet in vascular smooth muscle cells, Akt can directly phosphorylate HDAC5 upon stimulation by insulin-like growth factor-1 [35]. In its non-phosphorylated form, HDAC5 interacts with the transcription factor myocyte enhancer factor-2 (MEF2), which results in repression of its transcriptional activity, involved in hypertrophic growth [36,37]. Hence, Akt-stimulated HDAC5 phosphorylation may activate the MEF2-controlled hypertrophic program. Taken together, both Akt/mTORc1- and Akt/HDAC5-pathways may be necessary for PE-induced hypertrophy. On the other hand, several studies have reported a role for the PKD1-pathway in relation to cardiac hypertrophy [38,39]. HDAC5 is known to be one of the downstream targets of PKD1. Twenty-four-hour PE treatment indeed activated the PKD1-pathway, including the phosphorylation of HDAC5, indicating that the PKD1-pathway might also be involved in the PE-induced hypertrophic response. A causal relationship, however, remains to be elucidated. 

Cells stimulated with PE showed increased glucose uptake rates, a finding that matches with the characteristics of cardiac hypertrophy. At the signaling level towards GLUT4 translocation, we observed an increase in the phosphorylation levels of Akt and of AS160. The inhibitory effect of the Akt-I compound on glucose uptake confirms the involvement of this pathway in PE-induced glucose uptake. Furthermore, we found increased phosphorylation of PKD1 as evidenced by increased phosphorylation of its downstream target TropI. PKD1 is part of a distinct pathway involved in glucose uptake, independent from the Pi3K/Akt/AS160-pathway [22]. Whether PKD1 also contributes to the PE-induced increase in glucose uptake remains to be investigated. 

Interestingly, the increase in glucose uptake was already observed after 16 h PE treatment. At this time point, there were no changes yet in protein synthesis, hypertrophic markers, nor contractile function. This indicates that metabolic changes occur before any of the other hallmark changes of this in vitro model of cardiac hypertrophy. These findings are in line with in vivo PET-studies performed by Kundu et al. [40] and by Li et al. [7], who showed that changes in glucose uptake precede structural remodeling of the heart in mice undergoing transverse aortic constriction surgery and in spontaneous hypertensive rats, respectively. Based on these results, metabolic targeting may be a promising approach to improve cardiac outcome in hypertrophy and hypertrophy-induced heart failure. 

### 3.2. Metabolic Modulation Treatments 

**Figure 7:**
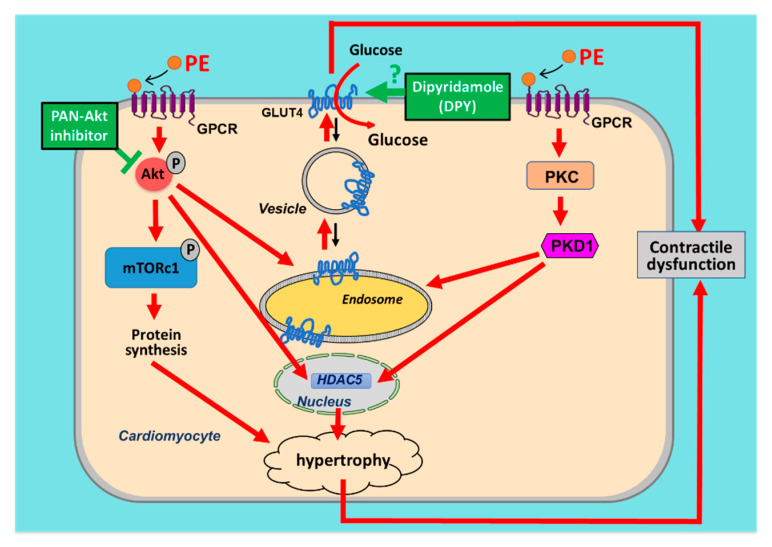
Integration of mechanisms of the phenylephrine (PE)-induced increase in glucose uptake and hypertrophy in cardiomyocytes—a hypothetical scheme. PE induces both activation of the Akt and protein kinase D (PKD1) signaling pathways. The PE-induced phosphorylation of Akt and of PKD1 leads to increased translocation of the main glucose transporter GLUT4 from the endosomes to the cell surface, resulting in increased glucose uptake. At the same time, activated Akt and PKD1 lead to the phosphorylation and nuclear export of HDAC5, resulting in release and activation of hypertrophic transcription factors. Additionally, PE-induced Akt phosphorylation causes activation of mTOR, a major regulator of protein synthesis. Both the PE- induced increase in glucose uptake and hypertrophy contribute to contractile alterations. Treatment of cardiomyocytes with PAN-Akt inhibitor or DPY inhibit GLUT4 translocation or GLUT4-mediated glucose transport, respectively, and protect the cell from contractile alterations. Since DPY treatment does not restore the PE-induced increases in protein synthesis and hypertrophic markers, yet restores contractile properties, it follows that targeting metabolism, independent from hypertrophy, forms a promising strategic approach to prevent hypertrophy-induced contractile dysfunction. The arrows indicate the directions. The -–I symbol indicates inhibition.

The importance of substrate metabolism in the development of hypertrophy and hypertrophy-induced heart failure was confirmed in the present study by the use of compounds with the ability to alter metabolism. Both Akt-I and DPY decreased PE-stimulated glucose uptake, thereby shifting metabolism away from glucose utilization. The fact that only Akt-I was able to prevent the PE-induced increase in hypertrophic markers and protein synthesis, probably through direct links of Akt with HDAC5 [35] and mTOR, while both compounds were able to restore contractile function, indicates that the metabolic alterations upon development of cardiac hypertrophy are most likely of greater relevance in the development of cardiac dysfunction (Figure 7). 

That DPY is able to improve contractile function was shown before in an in vivo study where aortic banded rats were treated with DPY [41]. In these DPY-treated rats, left ventricular filling abnormalities were prevented, responsiveness to isoproterenol was preserved, and detrimental chamber remodeling was attenuated compared to that of saline treated aortic banded rats. Interestingly, the positive effects on cardiac function in these DPY-treated rats were not associated with a reduction in relative wall thickness, so these rats still developed cardiac hypertrophy [41]. This finding matches with the observation in our study that DPY was not able to reduce expression levels of hypertrophic genes or able to affect protein synthesis. Hence, inhibition of glucose uptake by DPY preserves contractile activity under hypertrophic stimulation but does not prevent the maladaptive increase in protein synthesis. In addition, another GLUT4 inhibitor, ritonavir, did not inhibit PE-induced protein synthesis (Supplementary Figure S3), suggesting that this beneficial effect of DPY does not involve reduction of hypertrophy. 

The mechanism by which DPY exerts its effects on contractile function remains unclear. It is expected that, like with Akt-I, the inhibition of GLUT4-mediated glucose uptake is involved. Furthermore, because DPY did not decrease phosphorylation of Akt, AS160, PKD1, nor Trop1 we may conclude that the Pi3K/Akt/AS160-pathway and the PKD1-pathway are not involved. Several studies have shown that repressing glycolysis during hypertrophic stimulation is beneficial for the heart [11,21]. In agreement with this, our current data suggests that inhibition of GLUT4-mediated glucose uptake (either via blocking GLUT4 translocation or blocking GLUT4 transport activity) prevents hypertrophy-induced contractile alterations. The underlying molecular mechanism of why increased glucose uptake is detrimental remains to be elucidated, but it is to be expected that inhibition of glucose uptake prevents (i) maladaptive O-GlcNAcylation [42], (ii) a shift to expression of neonatal isoforms of contractile proteins (thereby negatively influencing contraction dynamics [43]), and/or (iii) accumulation of toxic glycolytic intermediates [40]. 

Further research is required to unravel this latter mechanism. Nevertheless, both Akt-I and DPY improved contractile function in PE-stimulated cells, indicating that metabolic targeting forms a promising strategic approach to prevent cardiac dysfunction in the hypertrophic heart. 

## 4. Materials and Methods 

### 4.1. Animals

Ten 15-week-old male Lewis rats (300–400 g) were purchased from Charles River Laboratories. Animals were housed at the Experimental Animal Facility of Maastricht University (The Netherlands) in a temperature- and humidity-controlled environment subjected to a 12 h light/dark cycle and free access to food and water.

### 4.2. Isolation and Treatment of Adult Rat Cardiomyocytes

Rats were anaesthetized intraperitoneally with pentobarbital (200 mg/kg) after which the hearts were rapidly removed. Adult rat cardiomyocytes (aRCM) were isolated by Langendorff perfusion as previously described [44], seeded on laminin-coated plates (Sigma, St. Louis, MO, USA), and cultured in M199 culture medium (Gibco 31153, Thermo Fischer, Waltham, MA, USA) supplemented with 5 mM creatine, 3.2 mM carnitine, 3.1 mM taurine, 20 μM palmitate, and 1% penicillin/streptomycin. Cells used for the time course experiments were cultured in the presence or absence of 50 μM PE (Sigma, St. Louis, MO, USA) for 16 h, 24 h, or 48 h. The isolation yielded 80–90% normal, rod-shaped cardiomyocytes with no difference between control and PE-stimulated cells. For cell size measurements, cells were segmented manually and size was measured with FIJI [45]. Images were acquired with an inverted microscope with a 20× objective phase contrast mode. For autophagy measurements, control and PE-stimulated cells were co-treated with 100 nM bafilomycin (a compound known to inhibit autophagy by targeting lysosomes). Cells used for metabolic intervention experiments were either pretreated with 10 μM DPY (Sigma, St. Louis, MO, USA) for 2 h followed by 22 h of 50 μM PE and 10 μM DPY co-treatment or cultured with or without 50 μM PE in the presence or absence of 5 μM Akt-I (Sigma, St. Louis, MO, USA) for 24 h. 

### 4.3. RNA Isolation and RT-PCR

Total RNA was isolated using Tri Reagent (Sigma, St. Louis, MO, USA) and 1 µg cDNA was synthesized using the SensiFAST cDNA synthesis kit (Bioline, GC Biotech, Waddinxveen, The Netherlands). Relative gene expression was determined by RT-PCR using Sensimix SYBR and Fluorence kit (Bioline, GC Biotech, The Netherlands) and the ABI 7900HT Fast Real-Time PCR System. The following genes were analyzed (5′ → 3′): ANF (forward: CCTCTTCCTGGCCTTTTGG; reverse: CCAGGTGGTCTAGCAGGTTCTT) BNP (forward: AGGAGAGACTTCGAAATTCCAAGA; reverse: CTAAAACAACCTCAGCCCGTCA). The ΔΔCT method was used for quantification and samples were normalized against the housekeeping gene Cyclophilin A (forward: TTCCTCCTTTCACAGAATTATTCCA; reverse: CCGCCAGTGCCATTATGG).

### 4.4. Immunoblotting

Equal volumes of samples were loaded and proteins were separated by SDS-PAGE and transferred to nitrocellulose membrane for Western blotting. Antibodies against LC3B, p62, phosphorylated ACCSer79 (pACC), phosphorylated AktSer473 (pAkt), phosphorylated AMPKThr172 (pAMPK), total AMPK, phosphorylated AS160Thr642 (pAS160), phosphorylated ERK1/2Thr202/Tyr204, phosphorylated HDAC5Ser498 (pHDAC5), phosphorylated mTORSer2448 (pmTOR), total mTOR, phosphorylated PKD1Ser744/748 (pPKD744), phosphorylated PKD1Ser916 (pPKD916), phosphorylated rS6Ser235/236 (prS6), phosphorylated TropISer23/24 (pTropI), and phosphorylated 4EBPThr37/46 (p4EBP) were used for detection. LC3B, p62, pAkt, pAMPK, pAS160, pmTOR, pPKD744, pPKD916, prS6, pTropI, p4EBP, and β-actin were obtained from Cell Signaling (Danvers, MA, USA). The antibody directed against pACC was from Upstate (Dundee, UK), pHDAC5 from Abcam (Cambridge, UK), and CAV3 from BD transduction Laboratories (San Jose, CA, USA). Membranes were blocked with 5% non-fat dry milk or 5% BSA in Tris-buffered saline with 0.1% Tween, incubated with primary antibodies overnight, and washed prior to incubation with HRP-conjugated secondary antibodies. Samples were normalized against the loading controls caveolin-3 (CAV3) or β-actin (β-actin). The protein bands were visualized using enhanced chemiluminescence (ClarityTM Western, Biorad, Hercules, CA, USA). 

### 4.5. Substrate Uptake into Cardiomyocytes

Deoxyglucose uptake was measured as previously described [45]. In short, cells, cultured on glass coverslips, were washed and a mixture deoxy-D-glucose (4 µM) and tracer amounts of radioactive-labelled [1-3H]-deoxyglucose (0.217 µCi/mL, Perkin Elmer, Waltham, MA, USA) was added. After 10 min incubation, the uptake was stopped by addition of ice-cold wash buffer containing 0.2 M phloretin (Sigma, St. Louis, MO, USA). Cells were lysed in 1 M NaOH and radioactivity was measured with a β-counter.

### 4.6. GLUT4 Translocation in Cardiomyocytes

Adenoviral construction expressing hemagglutinin (HA)-GLUT4-green fluorescent protein (GFP) was kindly provided by Prof. Luc Bertrand (Institute of Experimental and Clinical Research (IREC), Pole of Cardiovascular Research, UCLouvain, Brussels, Belgium). Evaluation of GLUT4 translocation was conducted as previously described with the modifications described below [46,47]. Briefly, GFP was fused to the carboxyl-terminus of GLUT4 to track all exogenous GLUT4. In contrast, the HA epitope was inserted in the first exofacial loop of GLUT4, allowing the exclusive detection of GLUT4 inserted into the membrane of non-permeabilized cells. aRCMs were infected with HA-GLUT4-GFP adenoviruses at a multiplicity of infection of 5 for 48 h. After 3.7% formaldehyde fixation and blocking with 5% BSA, non-permeabilized cells were incubated with an anti-HA-tag primary antibody (Cell Signaling Technology, Danvers, MA, USA), followed by a fluorescent secondary antibody (Alexa Fluor 594) (Invitrogen, Carlsbad, CA, USA). Nuclei were counterstained with DAPI. The cells were mounted on glass slides and imaged at 63x objective with a confocal microscope (Leica SPE). For increased visibility, equal linear adaptation of brightness/contrast was applied and recordings were converted to 8-bit images using ImageJ/FIJI software (version 1.53c).

### 4.7. Protein Synthesis

Cultured cells were treated with [3H]-phenylalanine (0.1 μCi/mL) in the presence or absence of PE for 16 h, 24 h, 48 h, and in selected experiments with either PE+Akt-I or PE+DPY for 24 h. After incubation cells were washed three times with ice-cold 1× PBS, and proteins were precipitated in 10% trichloroacetic acid (Sigma, St. Louis, MO, USA) overnight (4 °C). The samples were then briefly rinsed two times with 95% ethanol, scraped in 1 M NaOH, and radioactivity was measured via scintillation counting with a β-counter.

### 4.8. Cardiomyocyte Contractile Function

Contractile properties of cardiomyocytes were assessed as previously described [48]. Sarcomere dynamics were measured at 1 Hz electric field stimulation using video and Fast Fourier Transform based sarcomere length detection (IonOptix, Amsterdam, The Netherlands). Sarcomere shortening, peak rate, and time to peak and decay time were calculated from digitized recordings acquired with IonWizard acquisition software. 

### 4.9. Statistics

Data are presented as means ± standard deviation (STDEV). Comparisons between two groups with normally distributed data were performed with a 2-tailed paired Student’s-*t*-test. In case of not-normally distributed data, the Wilcoxon signed-rank test was applied in the statistical analysis software Prism 5 (GraphPad Software, Inc., San Diego, CA, USA). For comparisons of >2 groups, a two-way ANOVA was used followed by post-hoc testing with Bonferroni correction in the IBM SPSS statistics version 24.0 (IBM, Corp., Armonk, NY, USA). Statistical significance was set at *p* < 0.05.

## 5. Conclusions

Taken together, PE stimulation of adult rat cardiomyocytes exhibits the main characteristics (increased glucose uptake, increased protein synthesis, increased expression of hypertrophic markers, and reduced contractile function) of cardiac hypertrophy and hypertrophy-induced heart failure over time, which will allow us in the future to study metabolic changes that trigger the development of hypertrophy and hypertrophy-induced heart failure in more detail. Interestingly, by applying this model of hypertrophy and hypertrophy-induced heart failure we established that metabolic changes precede structural and functional changes. As a corollary, targeting metabolism with or without simultaneously targeting protein synthesis, by pharmacological interventions aimed at reducing myocardial glucose uptake, forms a valuable strategy to prevent cardiac dysfunction. Future studies should focus on the translation of these in vitro findings towards an in vivo setting. Moreover, other compounds that decrease cardiac glucose uptake might be of high value in designing novel strategies to combat hypertrophy-induced heart failure. 

## Data Availability

The data presented in this study are available on request from the corresponding author.

## Supplementary Materials

The supplementary materials are available online at https://www.mdpi.com/article/10.3390/ijms22073620/s1.

## Abbreviations

PE	Phenylephrine
DPY	Dipyridamole
Akt-I	PAN-Akt inhibitor
ANF	Atrial natriuretic factor
BNP	Brain natriuretic peptide
Pi3K	Phosphatidylinositol-4,5-bisphosphate 3-kinase
PKB/Akt	Protein kinase B
mTOR	Mammalian target of rapamycin
FAT/CD36	Fatty acid translocase/cluster of differentiation 36
aRCM	Adult rat cardiomyocytes
CAV3	Caveolin 3
ET-1	Endothelin 1
Ang-II	Angiotensin II
mTORC1	Mammalian target of rapamycin complex 1
4EBP	4E-binding
protein rS6	Ribosomal protein S6
HDAC5	Histone deacetylase 5
PKD1	Protein kinase-D 1
AMPK	AMP-activated protein kinase
TropI	Troponin I
ACC	Acetyl-CoA carboxylase
MEF2	Myocyte enhancer factor-2
PDE	Phosphodiesterase
GLUT4	Glucose transporter 4
PKA	Protein kinase A

## References

[1] Savarese G., Lund L.H. (2017). Global Public Health Burden of Heart Failure. Card Fail. Rev..

[2] Levy D., Garrison R.J., Savage D.D., Kannel W.B., Castelli W.P. (1990). Prognostic implications of echocardiographically determined left ventricular mass in the Framingham Heart Study. N. Engl. J. Med..

[3] Koren M.J., Devereux R.B., Casale P.N., Savage D.D., Laragh J.H. (1991). Relation of left ventricular mass and geometry to morbidity and mortality in uncomplicated essential hypertension. Ann. Intern. Med..

[4] Frey N., Katus H.A., Olson E.N., Hill J.A. (2004). Hypertrophy of the heart: A new therapeutic target?. Circulation.

[5] Stanley W.C., Recchia F.A., Lopaschuk G.D. (2005). Myocardial substrate metabolism in the normal and failing heart. Physiol. Rev..

[6] Karwi Q.G., Uddin G.M., Ho K.L., Lopaschuk G.D. (2018). Loss of Metabolic Flexibility in the Failing Heart. Front. Cardiovasc. Med..

[7] Li J., Kemp B.A., Howell N.L., Massey J., Mińczuk K., Huang Q., Chordia M.D., Roy R.J., Patrie J.T., Davogustto G.E. (2019). Metabolic Changes in Spontaneously Hypertensive Rat Hearts Precede Cardiac Dysfunction and Left Ventricular Hypertrophy. J. Am. Heart Assoc..

[8] Dong M., Ding W., Liao Y., Liu Y., Yan D., Zhang Y., Wang R., Zheng N., Liu S., Liu J. (2015). Polydatin prevents hypertrophy in phenylephrine induced neonatal mouse cardiomyocytes and pressure-overload mouse models. Eur. J. Pharmacol..

[9] Fuller S.J., Gaitanaki C.J., Sugden P.H. (1990). Effects of catecholamines on protein synthesis in cardiac myocytes and perfused hearts isolated from adult rats. Stimulation of translation is mediated through the alpha 1-adrenoceptor. Biochem. J..

[10] Rolfe M., McLeod L.E., Pratt P.F., Proud C.G. (2005). Activation of protein synthesis in cardiomyocytes by the hypertrophic agent phenylephrine requires the activation of ERK and involves phosphorylation of tuberous sclerosis complex 2 (TSC2). Biochem. J..

[11] Ritterhoff J., Young S., Villet O., Shao D., Neto F.C., Bettcher L.F., Hsu Y.A., Kolwicz S.C., Raftery D., Tian R. (2020). Metabolic Remodeling Promotes Cardiac Hypertrophy by Directing Glucose to Aspartate Biosynthesis. Circ. Res..

[12] Dorn G.W., Force T. (2005). Protein kinase cascades in the regulation of cardiac hypertrophy. J. Clin. Investig..

[13] Aoyagi T., Matsui T. (2011). Phosphoinositide-3 kinase signaling in cardiac hypertrophy and heart failure. Curr. Pharm. Des..

[14] Prasad A.M., Inesi G. (2009). Effects of thapsigargin and phenylephrine on calcineurin and protein kinase C signaling functions in cardiac myocytes. Am. J. Physiol. Cell Physiol..

[15] Zhang C.L., McKinsey T.A., Chang S., Antos C.L., Hill J.A., Olson E.N. (2002). Class II histone deacetylases act as signal-responsive repressors of cardiac hypertrophy. Cell.

[16] Mori J., Basu R., McLean B.A., Das S.K., Zhang L., Patel V.B., Wagg C.S., Kassiri Z., Lopaschuk G.D., Oudit G.Y. (2012). Agonist-induced hypertrophy and diastolic dysfunction are associated with selective reduction in glucose oxidation: A metabolic contribution to heart failure with normal ejection fraction. Circ. Heart Fail..

[17] Fischer Y., Böttcher U., Eblenkamp M., Thomas J., Jüngling E., Rösen P., Kammermeier H. (1997). Glucose transport and glucose transporter GLUT4 are regulated by product(s) of intermediary metabolism in cardiomyocytes. Biochem. J..

[18] Herrero P., Sharp T.L., Dence C., Haraden B.M., Gropler R.J. (2002). Comparison of 1-(11)C-glucose and (18)F-FDG for quantifying myocardial glucose use with PET. J. Nucl. Med..

[19] Liang M., Jin S., Wu D.D., Wang M.J., Zhu Y.C. (2015). Hydrogen sulfide improves glucose metabolism and prevents hypertrophy in cardiomyocytes. Nitric Oxide.

[20] Sansbury B.E., Riggs D.W., Brainard R.E., Salabei J.K., Jones S.P., Hill B.G. (2011). Responses of hypertrophied myocytes to reactive species: Implications for glycolysis and electrophile metabolism. Biochem. J..

[21] Kolwicz S.C., Olson D.P., Marney L.C., Garcia-Menendez L., Synovec R.E., Tian R. (2012). Cardiac-specific deletion of acetyl CoA carboxylase 2 prevents metabolic remodeling during pressure-overload hypertrophy. Circ. Res..

[22] Dirkx E., Schwenk R.W., Coumans W.A., Hoebers N., Angin Y., Viollet B., Bonen A., van Eys G.J., Glatz J.F., Luiken J.J. (2012). Protein kinase D1 is essential for contraction-induced glucose uptake but is not involved in fatty acid uptake into cardiomyocytes. J. Biol. Chem..

[23] Jeong M.Y., Walker J.S., Brown R.D., Moore R.L., Vinson C.S., Colucci W.S., Long C.S. (2010). AFos inhibits phenylephrine-mediated contractile dysfunction by altering phospholamban phosphorylation. Am. J. Physiol. Heart Circ. Physiol..

[24] Lionetti V., Stanley W.C., Recchia F.A. (2011). Modulating fatty acid oxidation in heart failure. Cardiovasc. Res..

[25] Glatz J.F.C., Nabben M., Young M.E., Schulze P.C., Taegtmeyer H., Luiken J.J.F.P. (2020). Re-balancing cellular energy substrate metabolism to mend the failing heart. Biochim. Biophys. Acta Mol. Basis Dis..

[26] Guo Y., Wang Z., Qin X., Xu J., Hou Z., Yang H., Mao X., Xing W., Li X., Zhang X. (2018). Enhancing fatty acid utilization ameliorates mitochondrial fragmentation and cardiac dysfunction via rebalancing optic atrophy 1 processing in the failing heart. Cardiovasc. Res..

[27] Steinbusch L.K., Schwenk R.W., Ouwens D.M., Diamant M., Glatz J.F., Luiken J.J. (2011). Subcellular trafficking of the substrate transporters GLUT4 and CD36 in cardiomyocytes. Cell Mol. Life Sci..

[28] Luiken J.J., Glatz J.F., Neumann D. (2015). Cardiac contraction-induced GLUT4 translocation requires dual signaling input. Trends Endocrinol. Metab..

[29] Sun A., Papur O.S., Dirkx E., Wong L., Sips T., Wang S., Strzelecka A., Nabben M., Glatz J.F.C., Neumann D. (2021). Phosphatidylinositol 4-kinase IIIβ mediates contraction-induced GLUT4 translocation and shows its anti-diabetic action in cardiomyocytes. Cell Mol. Life Sci..

[30] De Schryver E.L., Algra A., van Gijn J. (2003). Cochrane review: Dipyridamole for preventing major vascular events in patients with vascular disease. Stroke.

[31] MacWalter R.S., Shirley C.P. (2002). A benefit-risk assessment of agents used in the secondary prevention of stroke. Drug Saf..

[32] Shuralyova I., Tajmir P., Bilan P.J., Sweeney G., Coe I.R. (2004). Inhibition of glucose uptake in murine cardiomyocyte cell line HL-1 by cardioprotective drugs dilazep and dipyridamole. Am. J. Physiol. Heart Circ. Physiol..

[33] Luiken J.J., Coort S.L., Willems J., Coumans W.A., Bonen A., Glatz J.F. (2004). Dipyridamole alters cardiac substrate preference by inducing translocation of FAT/CD36, but not that of GLUT4. Mol. Pharmacol..

[34] Harvey P.A., Leinwand L.A. (2011). The cell biology of disease: Cellular mechanisms of cardiomyopathy. J. Cell Biol..

[35] Pietruczuk P., Jain A., Simo-Cheyou E.R., Anand-Srivastava M.B., Srivastava A.K. (2019). Protein kinase B/AKT mediates insulin-like growth factor 1-induced phosphorylation and nuclear export of histone deacetylase 5 via NADPH oxidase 4 activation in vascular smooth muscle cells. J. Cell Physiol..

[36] McKinsey T.A., Olson E.N. (2004). Cardiac histone acetylation—Therapeutic opportunities abound. Trends Genet..

[37] Simsek Papur O., Sun A., Glatz J.F.C., Luiken J.J.F.P., Nabben M. (2018). Acute and Chronic Effects of Protein Kinase-D Signaling on Cardiac Energy Metabolism. Front. Cardiovasc. Med..

[38] Avkiran M., Rowland A.J., Cuello F., Haworth R.S. (2008). Protein kinase d in the cardiovascular system: Emerging roles in health and disease. Circ. Res..

[39] Zhao D., Wang W., Wang H., Peng H., Liu X., Guo W., Su G., Zhao Z. (2017). PKD knockdown inhibits pressure overload-induced cardiac hypertrophy by promoting autophagy via AKT/mTOR pathway. Int. J. Biol. Sci..

[40] Kundu B.K., Zhong M., Sen S., Davogustto G., Keller S.R., Taegtmeyer H. (2015). Remodeling of glucose metabolism precedes pressure overload-induced left ventricular hypertrophy: Review of a hypothesis. Cardiology.

[41] Chung E.S., Perlini S., Aurigemma G.P., Fenton R.A., Dobson J.G., Meyer T.E. (1998). Effects of chronic adenosine uptake blockade on adrenergic responsiveness and left ventricular chamber function in pressure overload hypertrophy in the rat. J. Hypertens..

[42] Mailleux F., Gélinas R., Beauloye C., Horman S., Bertrand L. (2016). O-GlcNAcylation, enemy or ally during cardiac hypertrophy development?. Biochim. Biophys. Acta.

[43] Taegtmeyer H., Sen S., Vela D. (2010). Return to the fetal gene program: A suggested metabolic link to gene expression in the heart. Ann. N. Y. Acad. Sci..

[44] Luiken J.J., van Nieuwenhoven F.A., America G., van der Vusse G.J., Glatz J.F. (1997). Uptake and metabolism of palmitate by isolated cardiac myocytes from adult rats: Involvement of sarcolemmal proteins. J. Lipid Res..

[45] Schindelin J., Arganda-Carreras I., Frise E., Kaynig V., Longair M., Pietzsch T., Preibisch S., Rueden C., Saalfeld S., Schmid B. (2012). Fiji: An open-source platform for biological-image analysis. Nat. Methods.

[46] Schwenk R.W., Dirkx E., Coumans W.A., Bonen A., Klip A., Glatz J.F., Luiken J.J. (2010). Requirement for distinct vesicle-associated membrane proteins in insulin- and AMP-activated protein kinase (AMPK)-induced translocation of GLUT4 and CD36 in cultured cardiomyocytes. Diabetologia.

[47] Renguet E., Ginion A., Gélinas R., Bultot L., Auquier J., Robillard Frayne I., Daneault C., Vanoverschelde J.L., Des Rosiers C., Hue L. (2017). Metabolism and acetylation contribute to leucine-mediated inhibition of cardiac glucose uptake. Am. J. Physiol. Heart Circ. Physiol..

[48] Angin Y., Steinbusch L.K., Simons P.J., Greulich S., Hoebers N.T., Douma K., van Zandvoort M.A., Coumans W.A., Wijnen W., Diamant M. (2012). CD36 inhibition prevents lipid accumulation and contractile dysfunction in rat cardiomyocytes. Biochem. J..

