# Supplementary material for: Metabolic Interventions to Prevent Hypertrophy-Induced Alterations in Contractile Properties In Vitro
Source: Int J Mol Sci. 2021 Mar 31;22(7):3620. doi: 10.3390/ijms22073620 (PMC8037191; doi:10.3390/ijms22073620)
Supplement: Supplementary file 1 [file ijms-22-03620-s001.pdf]

Supplement

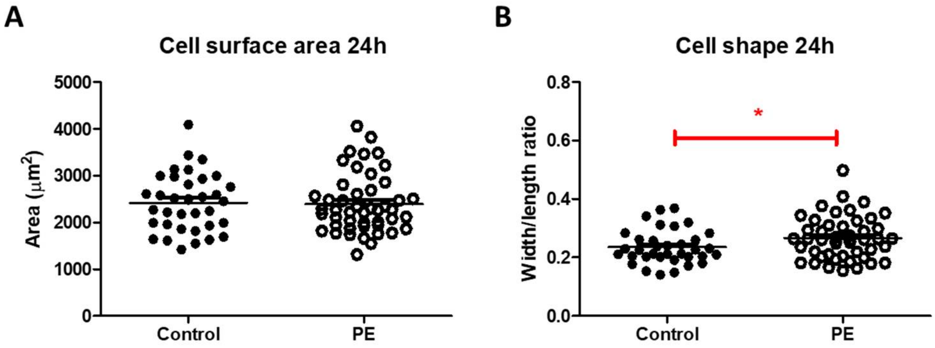

Supplementary figure 1. The effects of phenylephrine (PE) on cell size **(A)** and shape **(B)**. Cross-sectional area's (μm^2) **(A)** and cell width / length ratio **(B)** of ~40 cells from 3 different rats (~13 cells / rat) were measured in the presence or absence of 50 μM PE. Cells were segmented manually and size and shape were measured with FIJI. Images were acquired with an inverted microscope with a 20x objective phase contrast mode.

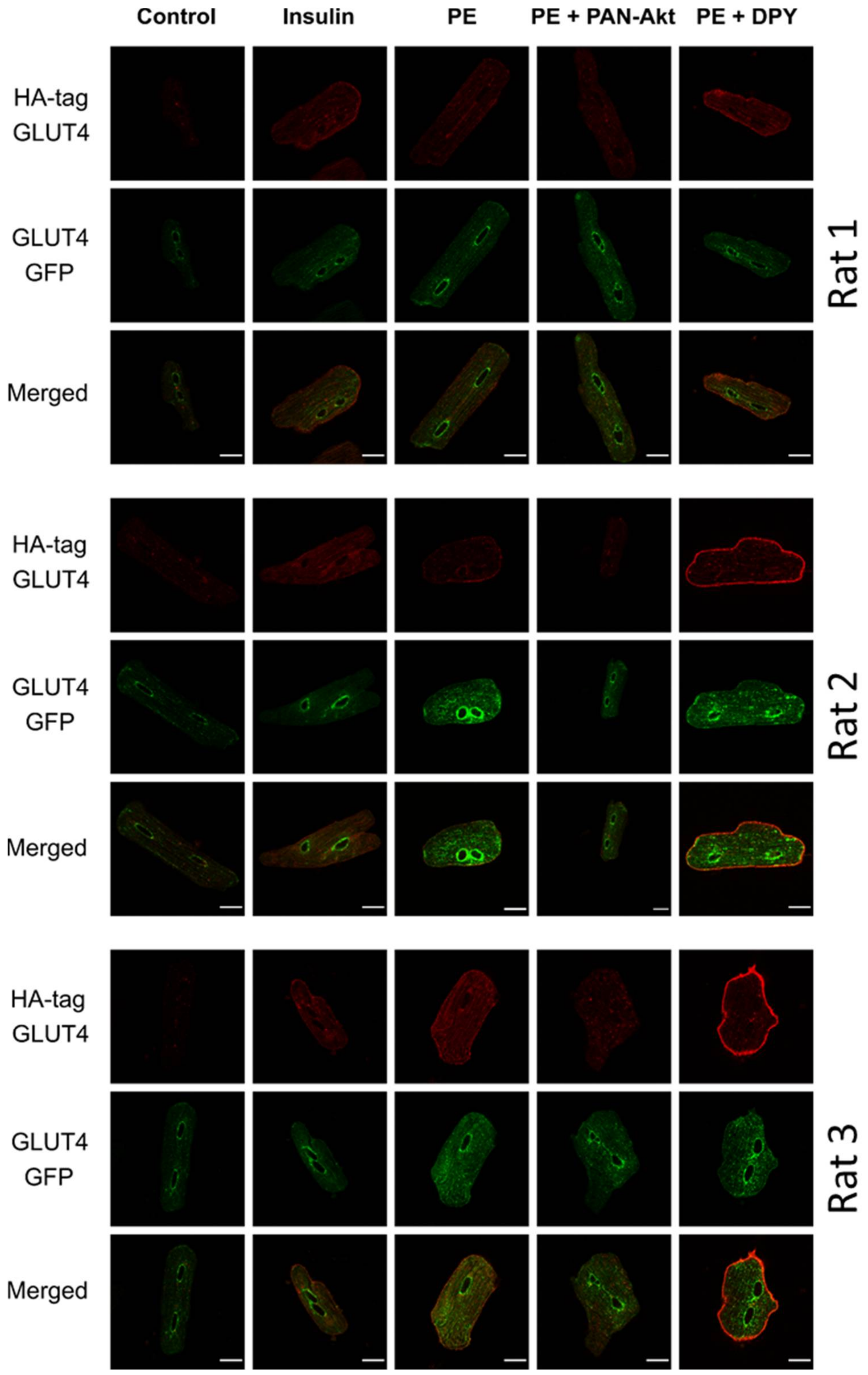

Supplementary figure 2. Representative confocal images of Glut4 translocation in cardiomyocytes from 3 different rats (scale bar 20 μm). The effect of phenylephrine (50 μM) stimulation in combination with / without PAN-Akt inhibitor (5 μM) or DPY (10 μM) on Glut4 translocation is shown. Insulin (100 nM, 30 minutes) was used as positive control.

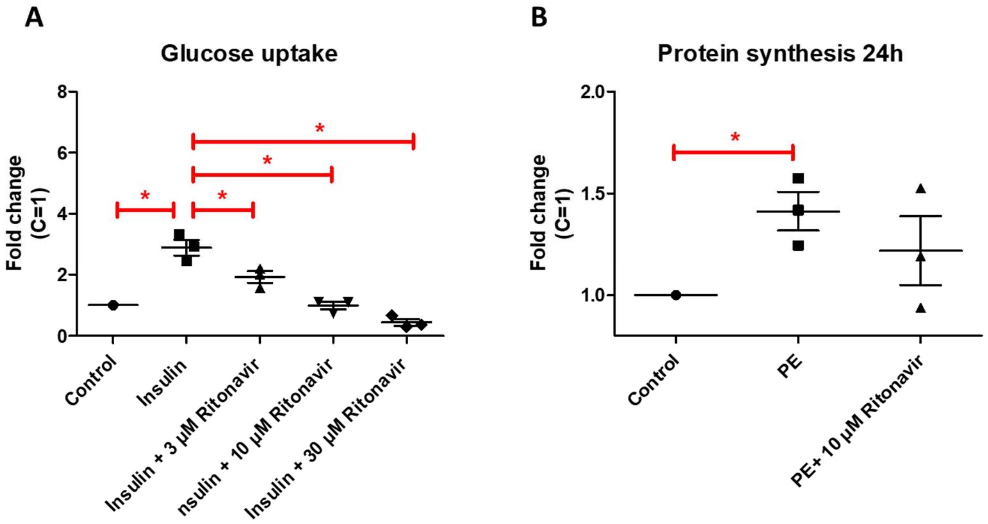

Supplementary figure 3. The effect of the GLUT-4 inhibitor, Ritonavir, on glucose uptake and protein synthesis. **A)** Glucose uptake after 15 minutes stimulation with 3 μM, 10 μM and 30 μM Ritonavir in the presence of insulin (100 μM). **B)** Protein synthesis after 24h stimulation with 10 μM Ritonavir in the presence of PE. Data are displayed in fold increase ± STDEV relative to control (n=3). *$p < 0.05$ was considered significantly different compared to control.
